# Estimating Emission Load from Road Transportation within the Bhaktapur Municipality, Nepal

**DOI:** 10.1155/2020/2828643

**Published:** 2020-11-06

**Authors:** Prasidha R. Neupane, Iswor Bajracharya, Bhai R. Manandhar, Meera Prajapati, Hishila Sujakhu, Pramod Awal

**Affiliations:** ^1^Khwopa College, Bhaktapur, Nepal; ^2^Nepal Academy of Science and Technology (NAST), Lalitpur, Nepal; ^3^SchEMS School of Environmental Science and Management, Kathmandu, Nepal

## Abstract

Vehicular emissions have been playing a pivotal role in deteriorating air quality in many urban parts of Nepal causing adverse impacts upon the health of commuters and pedestrians attributed to severe respiratory diseases. Primary data such as the number of vehicles (*N*) were obtained using two-hour peak (8 am to 10 am) and two-hour nonpeak (1 pm to 3 pm) count, after which average annual vehicle kilometer (VKT) and fuel economy (*F*) required for emission load estimation were obtained from vehicle survey using the simple random sampling method, sampling size taken statistically under 5% margin of error. Secondary data in this study include emission factors and derived equations from a published article. The vehicular emission load of Bhaktapur Municipality were found to be 3,310 tons/year including CO_2_, CO, NOx, HC, and PM_10_ of which CO_2_ accounts for 94.36% of total emissions followed by CO (4.39%), HC (0.72%), NOx (0.35%), and PM_10_ (0.18%), respectively. Significant positive correlation was found (*r* = 0.92, *p*=0.002) between CO_2_ and PM_10_ (*r* = 0.87, *p*=0.009), between CO_2_ and NOx (*r* = 0.90, *p*=0.004), between CO and HC (*r* = 0.74, *p*=0.05), and between NOx and PM_10_, respectively. The scenario analysis shows that the introduction of electric vehicles at different rates within the municipality can reduce the emissions to a significant amount. Exponential growth in vehicular gaseous pollutants potent to jeopardize the environment and welfare can become inevitable in the future if clean energy technology is not promoted early.

## 1. Introduction

Dust resuspension due to motor vehicles and construction, smoky vehicles, and the release of particulate matter (PM) by small-scale industries such as brick kilns is the major sources of air pollution [1]. Pollution emitted from vehicles is the function of fuel combustion reaction with other gases and atmospheric conditions [2]. Vehicular emissions originate from the interdependence of vehicle technology, fuel quality, and vehicle's condition of use [3]. The quality of fuel, both gasoline and diesel, conforms to the EuroIII standard in Nepal since 2010 [4] and is imported from India and overseas in the refined form for its direct consumption [5]. Vehicular emissions increase dramatically as the vehicle fleet increases along with variations in vehicle travel characteristics [6].

There is a strong link between air pollution and cardiovascular diseases such as strokes, heart disease, and cancer playing a significant role in the development of respiratory diseases [7]. Particles of 10 microns in diameter or less that is PM_10_ are responsible for irritation of eyes, nose, and acute respiratory infections [8], whereas PM_2.5_ or less than that has a high tendency to penetrate deep into the lungs or even dissolve in the bloodstream [9, 10]. In children and adults, PM_10_ is highly associated with mortality and respiratory illness [11]. Concentrations of ambient particulate matter to short-term and long-term exposure have increased the risk for cardiovascular events in humans [12]. Pedestrians are highly exposed to a high concentration of pollutants due to their proximity to vehicular emissions [13].

Over the last few decades, the impacts of vehicular emission on air pollution in Asian cities are more significant and have been gradually increasing [14]. Air pollution causes nearly 100,000 premature deaths per year and over a billion workdays of lost or reduced productivity in South Asian Cities [15]. The social costs of all environmental impacts from air pollution due to fuel consumption over six Asian cities were 3.8 billion US dollars of which health impacts costs were the highest of all [16]. The two largest cities, Beijing and Shanghai, regularly exceed emissions limit for multiple pollutants by double the safe amount recommended by WHO [15].

The amount of PM in Kathmandu Metropolitan city is significantly higher than the safe limits set by WHO [1]. In Kathmandu Valley, vehicles alone contribute about 38% of PM_10_ out of total particulate emission [17]. About 63% of total PM_10_ comes from vehicles and road dust, whereas resuspended dust contributes 25% of PM_10_ emission in Kathmandu valley [17]. The air quality of urban areas such as Kathmandu is poor causing a major impact on the health and welfare of the people and the environment [18]. The number of Chronic Obstructive Pulmonary Disease (COPD) patients increases often during the winter season when pollution is at peak [17, 19]. Institute for Health Matrix and Evaluation (IHME) ranked COPD in the second position as the most causative factor to trigger deaths in Nepal [20]. Study shows that 24,000 premature annual deaths are expected to happen in Nepal due to outdoor air pollution by 2030 [21].

## 2. Rationale of the Study

Few studies regarding vehicular emission [1, 22–26] has been carried out in Kathmandu valley, covering the entire area within the valley. However, this type of research has not yet been done in Nepal, focusing on a single municipality. This study focuses on the dynamics of road transport emitted pollutants potent to play a significant role in the deterioration of air quality of Bhaktapur Municipality that can be useful in facilitating further research in air pollution. This study can help formulate policy and guidelines to local authority in different ward levels of Bhaktapur Municipality useful for mitigating vehicle-induced pollution.

## 3. Materials and Methods

### 3.1. Sampling

**Figure 1:**
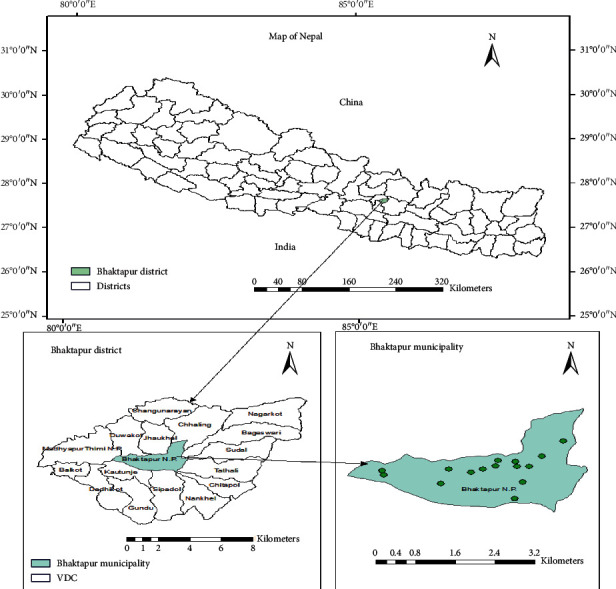
Map of the study area showing GPS points of sampling sites.

This study was conducted in the Bhaktapur Municipality, which lies to the east corner of Kathmandu valley, about 13 kilometers from the capital city Kathmandu. The Municipality has 10 administrative wards and occupies an area of 6.56 square kilometers with a population of 83,658 [27]. Fifteen GPS coordinates were generated from Earth explorer in a stratified random manner considering the road junction and traffic movement for the selection of sampling sites (Figure 1) prepared using GIS ArcMap 10.5. Data were collected in March 2018 from 15 coordinates located on the map of the Bhaktapur Municipality (Figure 1). The two-hour peak from 8 am to 10 am and two-hour nonpeak from 1 pm to 3 pm, in total 4 hours, were taken for the count of private vehicles, whereas recorded data of commercial vehicles were retrieved from its respective vehicle committee. After obtaining the total number of vehicles (by types) from the peak and nonpeak hour count, the sampling size for each vehicle (by types) was calculated statistically under a 95% confidence level and 5% margin of error. Due to the frequent movement of vehicles from Bhaktapur municipality to its adjacent city Kathmandu, the average annual vehicle kilometer (VKT) of each vehicle types was obtained by measuring the average aerial distance from each GPS points using ArcMap 10.5 to the main highway connecting both Kathmandu and Bhaktapur district multiplied by their average times of travel per day, data obtained from field survey taking different driving routes into account along with average fuel economy (Fi) of each vehicle (by types) (Table 1).

The primary data (Table 1) along with the help of secondary data (Table 2) adopted from the published article was used in the derived equations (1) and (2) to estimate the emission load of Bhaktapur Municipality.

Gaseous pollutants like carbon dioxide (CO_2_), carbon monoxide (CO), nitrogen oxides (NOx), hydrocarbon (HC), and particulate matter of 10 microns or less (PM_10_) were taken in the study as emission factors (Table 2) of those pollutants were available in a published article [1, 23].

Mathematical equations (see (1) and (2)) used in published articles [1, 22–24] were adopted for the estimation of vehicular emission load.

### 3.2. Equation 1: Equation for Annual Energy Demand Estimation

(1) $$\begin{array}{c} {\text{ED}}_{i,t}=N_{i,t}*{\text{VKT}}_{i,t}*F_{i}, \end{array}$$

where ED_*i,t*_ is the total annual energy demand in liters by vehicle type *i* in a year *t*. *N*_*i,t*_ is the total number of existing vehicles in year *t*. VKT_*i,t*_ is average annual mileage in kilometer. *F*_*i*_ is the average fuel economy in liters per kilometer.

### 3.3. Equation 2: Equation for Emission Load Estimation

(2) $$\begin{array}{c} E_{j,i,t}={\text{ED}}_{i,t}*{\text{EF}}_{j,i,t}, \end{array}$$

where *E*_*j,i,t*_ is the total emission of emission type *j* by vehicle type *i* in year *t*. ED_*t,i*_ is the total energy demand by vehicle type *i* in year *t*. EF_*j,i,t*_ is the emission factor of type *j* expressed in gram per liters of vehicle type *i* in year *t* (note that since (1) gives annual energy demand in liters, conversion factors for gasoline and diesel fuel were taken for conversion to energy equivalent in terms of Giga Joule [28]).

## 4. Results

Average annual energy demand was found to be 33,044 Giga Joule (GJ) of which mini truck accounts for 33% of total energy demand which is highest of all, followed by pickup van (18%), mini bus (17%), motorbike (16%), car/van (8%), others (7%), and mega bus (1%), respectively.

**Figure 2:**
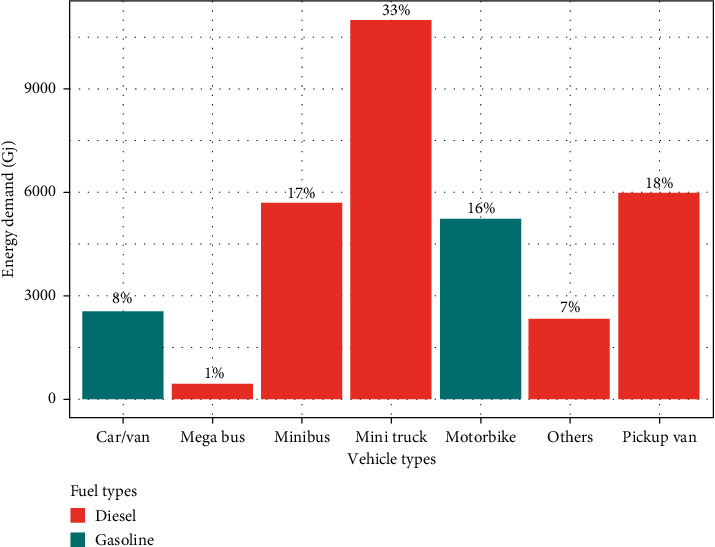
Average annual energy demand of each vehicle (by types) distinguished by fuel types.

**Figure 3:**
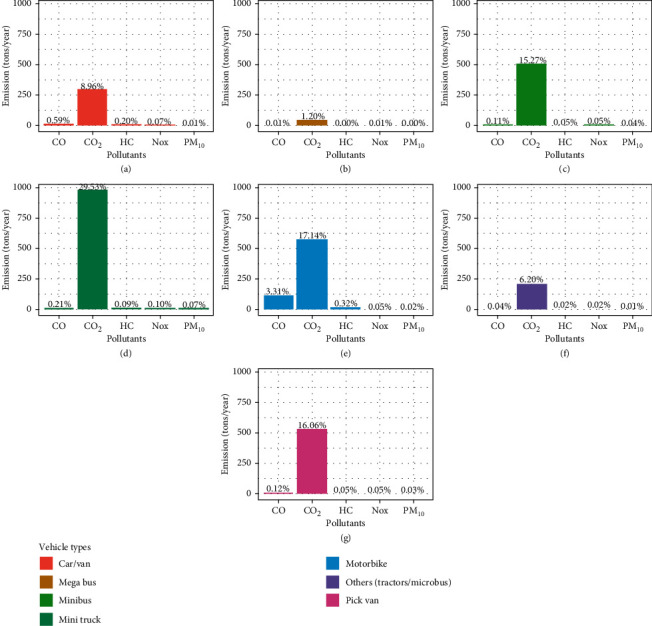
Vehicular emission load (by types) in percentages. (a) Car/van. (b) Mega bus. (c) Minibus. (d) Mini truck. (e) Motorbike. (f) Others (tractors/microbus). (g) Pickup van.

Mini truck was found to have the highest energy demand in terms of diesel fuel, whereas motorbike was found to have the highest energy demand in terms of gasoline fuel (Figure 2). The total emission load was estimated at 3310 tons/year of which CO_2_ was found to account for 94.36% of total emission followed by CO (4.39%), HC (0.72%), NOx (0.35%), and PM_10_ (0.18%), respectively. The CO_2_ was found to be the highest emitted pollutant and PM_10_ was found to be the least emitted pollutant and was found in trace amount in each vehicle (by types) (Figure 3) showing poor combustion of fuel. Since CO_2_ is used as the benchmark to calculate global warming potential (CO_2_e) of greenhouse gases, CO_2_ was the only greenhouse gas taken in our study which was estimated at 3,123 tons that equals to 850.95 tons of carbon [29].

Mini truck was found to have the highest emissions that account for 30% of the total emissions whereas mega bus was found to have the least contribution in emission that accounts for only 1% of the total emissions. Similarly, motorbike accounts for 21% of total emissions followed by pickup van and minibus (16%), car/van (10%), and others (6%), respectively (Figure 3). Mini truck was found to have the highest CO_2_ emission that accounts for 29.53% of total emissions followed by motorbike (17.14%), pickup van (16.06%), minibus (15.27%), car/van (8.96%), tractors/microbus (6.20%), and mega bus (1.20%), respectively (Figure 3). It shows that mega bus has a very small contribution towards CO_2_ emission even though these vehicles are larger in both size and engine capacity. Motorbike, on the other hand, was found to have the highest CO and HC emission that accounts for 3.31% and 0.32% of total emission followed by car/van (0.59%) CO and (0.20%) HC (Figure 3). Other pollutants like NOx and PM_10_ from vehicles (by types) were found in trace amounts.

**Figure 4:**
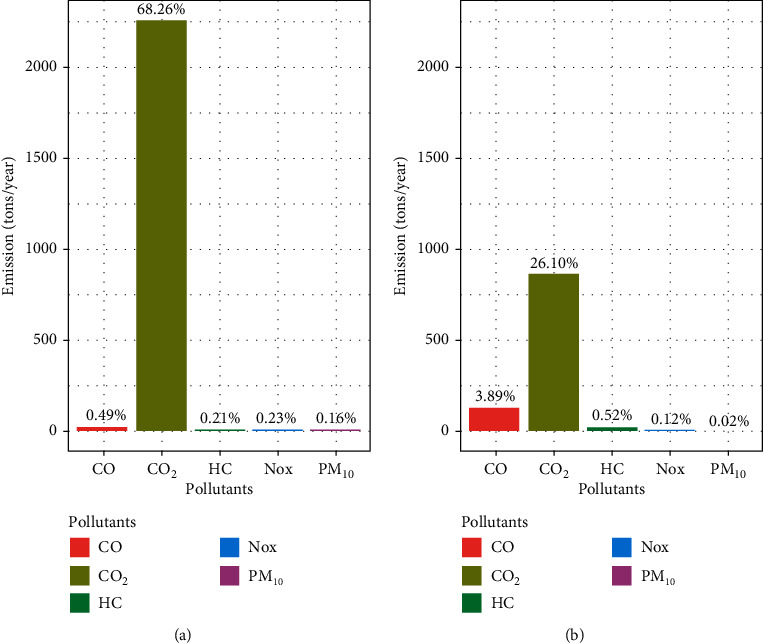
Share of pollutants (by fuel types) in percentages. (a) Diesel. (b) Gasoline.

The total emission from diesel fuel was found to be 2295 tons/year which is the highest compared to emission from gasoline fuel that was found to be 1015 tons/year. Diesel fuel was found to have a maximum share of CO_2_ emission accounting for 68.26% of total emissions followed by CO (0.49%), NOx (0.23%), HC (0.21%), and PM_10_ (0.16%), respectively. On the other hand, gasoline fuel was found to have a maximum share of CO_2_ emission accounting for 26.10% of the total emissions followed by CO (3.89%), HC (0.52%), NOx (0.12%), and PM_10_ (0.02%), respectively (Figure 4).

This study wants to reveal the potential changes in the total emissions which could be achieved by launching electric vehicles within the premises of Bhaktapur Municipality. This scenario analysis was done based on an assumption of the introduction of 3 electric vehicles (cars, motorbikes, and buses) at different rates (Table 3) which are considered feasible in Municipality. The different assumption rate for respective vehicles was multiplied with its original total number of respective vehicles from the study and calculated the reduced emissions through the same process followed above in our study. Total emission obtained from the reduced number of respective vehicles is then subtracted from the total emission estimated in our study to obtain a difference that can be achieved from lunching electric vehicles (Table 3). It is evident that if electric cars, motorbikes, and buses are introduced in the municipality by 10%, 20%, and 30% of the respective total number of vehicles plying on the Municipality road, a significant reduction in total emissions can be achieved.

Introducing electric vehicles at the rate of 10% of the respective total number of vehicles plying on the road, 156.84 tons of difference in total emission could be achieved in a year, whereas launching of electric vehicles at the rate of 20% and 30% of the respective total number of vehicles plying on the road, 313.68 tons and 470.52 tons of difference in total emissions could be achieved in a year, respectively. This could be significant progress to avert the risk of ambient air pollution if this initiative is taken at the earliest possible time from the concerned authority (local government).

**Figure 5:**
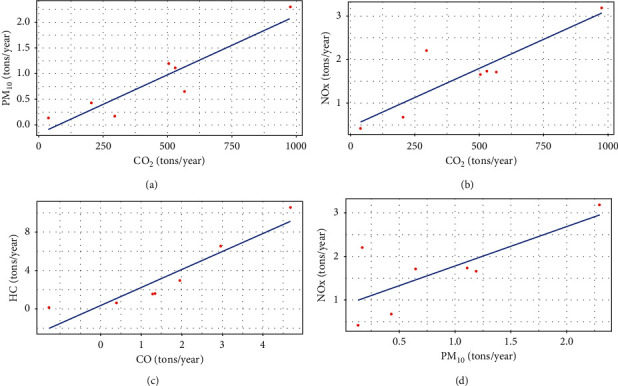
Scatter plot diagram showing the linear relationship: (a) between CO_2_ and PM_10_, (b) between CO_2_ and NOx, (c) CO and HC, and (d) between PM_10_ and NOx.

The correlation matrix (Table 4) shows both positive and negative correlations among pollutants under study. Pearson correlations shows the strong correlation between CO_2_ and PM_10_, CO_2_ and NOx, CO and HC, and NOx and PM_10_ (*r* = 0.92, *p*=0.002), (*r* = 0.87, *p*=0.009), (*r* = 0.90, *p*=0.004), (*r* = 0.74, *p*=0.05) Figures 5(a)–5(d).

Analysis on variance (ANOVA) test performed between PM_10_ and CO_2_(*p*=0.002) on regression equation (PM_10_ = −0.18 + 0.002 CO_2_) (Figure 5(a)), NOx and CO_2_(*p*=0.009) on the regression equation (NOx = 0.542 + 0.002 CO_2_) (Figure 5(b)), HC and CO (*p*=0.004) on the regression equation (HC = 1.61 + 0.086 CO) (Figure 5(c)), and NOx and PM_10_(*p*=0.05) on regression equation (NOx = 0.87 + 0.90 PM_10_) (Figure 5(d)) were found to be statistically significant (*p*=0.05). Scatter plot (Figures 5(a)–5(d)) showed an upbeat positive linear relationship between pollutants resulting in simultaneous increases in both study and explanatory variables.

## 5. Discussion

Taking the scenario of Kathmandu Metropolis, pollutant emission, which included CO, CO_2_, HC, NOx, SO_2_, TSP, and Pb, estimated in the year 2003 was 457,946 tons/year [23] and that is 139 times higher compared to the emission of Bhaktapur Municipality. In the year 2014, the total emission in Kathmandu Metropolis was 7231053.12 tons/year which included CO_2_, CO, NOx, HC, PM_10_, and SO_2_ of which CO_2_ accounts for 91% of the total emissions followed by CO (5.03%), HC (0.96%), NOx (0.60%), PM_10_ (0.18%), and SO_2_ (0.10%), respectively [24]. Emissions estimated between the year 2003 and the year 2014 for Kathmandu Metropolis showed unprecedented growth of emissions potent to affect its adjacent city such as Bhaktapur municipality, 7.35 times smaller than Kathmandu in a geographical area [23, 24]. Estimated emissions of Kathmandu in the year 2013 were 1,615,500 tons/year including (CO, VOC, NOx, PM, BC, and CO_2_) which was 4.5 times lower than the emission estimated in the year 2014; in other words, emission increased by five times in one year [24, 26]. There was an average growth rate of 14% in emissions from the year 2003 to 2014 [23, 26]. The number of vehicle fleet plays a crucial role in emissions. 70,000 vehicles were plying on the road of Kathmandu [24] which is 22 times higher than the vehicles plying on the road of Bhaktapur Municipality constituting 3,283 vehicles during our study in the year of 2018.

Taking the scenario of China, emission estimated in the year 2006 was 42,787,900 tons/year, whereas in the year 2010, it was estimated at 47,627,000 tons/year [30]. There was a growth rate of 2.8% per annum on average which is way less compared to an average emission growth rate in Kathmandu city between the years 2003 and 2013 [23, 26]. Similarly, in the year 2011, vehicular emissions of China were estimated at 44,679,100 tons/year in which CO was maximum by 32,700,000 tons/year and OC was minimum by 101500 tons/year [31]. The study revealed that emission decreased at the rate of −0.06% from 2010 to 2011, which was significant progress in terms of emission reduction [30, 31]. Emission studies in China for the years 1980–2005 estimated 674,629,000 tons/year of which CO_2_ was the highest in comparison to other pollutants. The previous study revealed that CO_2_ emission was almost the highest on a global scale [32].

Total CO_2_ emission estimated in our study was 3,123 tons/year which was much lesser than CO_2_ estimated in the year 2013 and year 2014 for Kathmandu Metropolis, comprising CO_2_ emission of 1,554,000 tons/year and 680,761.85 tons/year, respectively [24, 26]. Carbon dioxide was raised four times by the year of 2014 at Kathmandu. Several studies also showed that CO_2_ pollutants were higher compared to other pollutants [1, 22, 24, 26, 30–34].

Discussing emissions of particular pollutants from vehicles (by types), mini truck was found to emit the highest level of CO_2_ by 977.32 tons/year in our study, whereas, in the study of [24], bus/minibus was found to emit highest level of CO_2_ by 1,551,387.91 tons/year. However, the study also showed that private car/jeep/van contributed to the highest CO_2_ emission [1]. Similarly, in our study, motorbike was found to emit the highest level of CO by 109.37 tons/year and mega bus was found to emit the lowest level of CO by 0.28 tons/year, whereas, in the contrary, motorbike was found to emit the highest level of CO by 233,364.06 tons/year and tractors were found to emit the lowest level of CO by 90.10 tons/year in Kathmandu metropolis [24]. NOx and PM_10_ emission was found to be the highest in mini truck by 3.18 tons/year and 2.30 tons/year, respectively, while HC was found to be the highest in motorbike by 10.53 tons/year, whereas, in Kathmandu Metropolis, vehicles such as minibus, two-wheelers, and bus/minibus were found to emit the highest level of NOx, HC, and PM_10_ by 17,538.71 tons/year, 22,459.24 tons/year, and 5,764.13 tons/year, respectively [24].

In our study, diesel fuel was found to have a maximum share of CO_2_ by 68.26% and the minimum share of PM_10_ by 0.16%, whereas, in the contrary, diesel fuel was found to have a maximum share of PM_10_ by 38.2% and the minimum share of HC by 3.0% in Kathmandu [1]. On the other hand, gasoline fuel was found to have a maximum share of CO_2_ by 26.10% and the minimum share of PM_10_ by 0.02% in our study, whereas, in the contrary, gasoline fuel was found to have a maximum share of CO_2_ by 97.7% and the minimum share of HC by 3% in Kathmandu [1].

The total amount of emissions estimated for two adjacent neighboring cities contradicts here due to variation in spatiotemporal dimensions and vehicle fleet resulting in the need for different resolutions while formulating policy and guidelines to mitigate vehicles induced ambient pollution.

## 6. Conclusion

Diesel fuel was found to play a crucial role in emitting a high amount of greenhouse gas such as carbon dioxide leading to deteriorating air quality. Bhaktapur Municipality comprises an area of 6.56 square kilometers which is way much smaller compared to its adjacent city Kathmandu. There is a substantial disparity between the two neighboring cities in terms of population density, area size, and pollution so that any policy formulated and implemented concerning air pollution/control programs in Kathmandu valley needs to be customized to specific conditions of these two cities. Due to the smaller area size of Bhaktapur Municipality, the total emission from the transport sector, which stands at 3,310 tons/year, appears to be a small amount of emission contributed from the transport sector but this amount can play a significant role in air quality deterioration of this small area.

Replacement of fossil fuel with clean energy fuel such as compressed natural gas (CNG), Liquefied Natural Gas (LNG), Renewable Natural Gas (RNG), or technology relying upon perpetual energy source such as solar radiation is needed in the Bhaktapur Municipality to sustain human health, well-being, and welfare. Besides, emission control strategies should be fully implemented by the commercial industrial sector who are the major contributors to environmental pollution to regulate the optimum and efficient burning of fossil fuel. The formulation of policy, guidelines, and effective preparedness by local authorities along with robust solidarity resolute to mitigate air pollution with cohesive strategies of implementation is mandatory for the sustainable development of Bhaktapur Municipality.

## 7. Limitations

The authors of this study did not extend sampling time due to financial constraints and are limited to 4 hours/day for 1 month, adequate to reflect the tentative total road transport emissions of Bhaktapur Municipality. Due to the small spatial area, the data from this study do not generalize the whole Nation or province.

This study does not incorporate the latest emission factors published in the year of 2019 [35] nor did it address the relations between emission factors obtained during idling and running conditions of vehicles based on field measurement [25] potent to bring more accuracy in emission estimation to this study. Emission factors used in this study were static and do not consider its changes in nature due to surrounding environmental conditions, speed of vehicles, and age of vehicles.

## Figures and Tables

**Table 1:** Total number of vehicles (by types) plying on the road of Bhaktapur Municipality with their average annual vehicle kilometer and fuel economy.

Vehicle types	Fuel types	Total number of vehicles (N_*i,t*_)	Average annual vehicle kilometer (VKT_*i,t*_) in km	Fuel economy (F_i_) in l/km
Mega bus	Diesel	20	2016.26	0.28
Mini bus	Diesel	201	2923.58	0.25
Car/van	Gasoline	448	2243.09	0.07
Pickup van	Diesel	212	4738.21	0.15
Mini truck	Diesel	135	8417.89	0.25
Motor bike	Gasoline	2212	2893.33	0.02
Others (tractors/microbus)	Diesel	55	6502.44	0.16

**Table 2:** Emission factors (amount of pollutants emitted per unit distance traveled) given in gram per liters (g/l).

Vehicle types	Fuel types	CO_2_	CO	NOx	HC	PM_10_
Mega bus	Diesel	3440	24	35.61	11.1	11.7
Mini bus	Diesel	3440	24.8	11.2	10.4	8.1
Car/van	Gasoline	3985	261.9	29.6	87.9	2.27
Pickup van	Diesel	3440	24.8	11.2	10.4	7.2
Mini truck	Diesel	3440	24.8	11.2	10.4	8.1
Motor bike	Gasoline	3766	726.3	11.3	69.9	4.3
Others (tractors/microbus)	Diesel	3440	24.8	11.2	10.4	7.2

**Table 3:** Potential changes in total emissions due to introducing electric vehicle at different rates.

Introducing electric cars, motorbikes, and buses at different rates (%)	Emission estimated in our study (tons/year)	Emission after the launch of electric vehicles: buses, cars, and motorbikes (tons/year)	Difference (tons/year)
10	3309.31	3152.47	156.84
20		2995.63	313.68
30		2838.79	470.52

**Table 4:** Correlation matrix among various pollutants.

	CO_2_	CO	NOx	HC	PM_10_
CO_2_	1	—	—	—	—
CO	0.19	1	—	—	—
NOx	0.87^*∗*^	0.11	1	—	—
HC	0.27	0.90^*∗*^	0.38	1	—
PM_10_	0.92^*∗*^	−0.13	0.74^*∗*^	−0.10	1

## Data Availability

The data used to support the findings of this study are available from the corresponding author upon request.

## Supplementary Materials

Supplementary Materials The primary data collected from the field survey used for the estimation of vehicular emission load of Bhaktapur Municipality, Nepal, in our study are provided in supplemental files attested along with this manuscript archived in the journal of Environmental and Public Health (Hindawi). Moreover, the results data in table format will be provided upon the special request.
